# Vancomycin-soaked autografts during ACL reconstruction reduce the risk of post-operative infection without affecting return to sport or knee function

**DOI:** 10.1007/s00167-020-05879-9

**Published:** 2020-02-05

**Authors:** Yoann Bohu, Shahnaz Klouche, Hasan Basri Sezer, Serge Herman, Olivier Grimaud, Antoine Gerometta, Alain Meyer, Nicolas Lefevre

**Affiliations:** 1 Clinique du Sport Paris, 36 Boulevard Saint Marcel, 75005 Paris, France; 2 Racing 92, 11 Avenue du Plessis, 92350 Plessis-Robinson, France; 3 Institut de l’Appareil Locomoteur Nollet, 23, Rue Brochant, 75013 Paris, France; 4 ELSAN, 58bis Rue de la Boétie, 75008 Paris, France

**Keywords:** ACL reconstruction, Vancomycin, Return to sport, Comparative study, Functional results

## Abstract

**Purpose:**

To compare return to sport and knee function 1 year after anterior cruciate ligament (ACL) reconstruction using autografts with and without vancomycin presoaking.

**Methods:**

A case–control study based on a retrospective analysis of prospective data included athletes over the age of 16 operated from 2012 to 2018 for ACL reconstruction. There were two groups of patients due to a change in treatment protocols: Group 1 « without vancomycin » before November 2016 and Group 2 « with vancomycin» after this date. In Group 2, the graft was soaked in a vancomycin solution for 10 min and then fixed into the bone tunnels. The primary evaluation criterion was the return to sport 1 year after surgery. The secondary criteria were various knee scores. The number of patients needed to perform a non-inferiority study was calculated.

**Results:**

1674 patients fulfilled the selection criteria, 1184 in Group 1 and 490 in Group 2. The series included 1112 men and 562 women, mean age 30 ± 9.7 years, 68 professional athletes, 674 competitive athletes and 932 recreational athletes. While seven patients presented with post-operative septic arthritis in Group 1, this complication was not found in Group 2. No significant difference was identified in the return to running between the two groups 1 year after surgery (75.9% vs. 76.1%, n.s.). Significantly more of the patients in Group 2 returned to their preinjury sport (*p* = 0.04). Knee function was comparable between the groups.

**Conclusion:**

Vancomycin-soaked grafts during ACL reconstruction reduce the risk of post-operative infection of the knee without affecting the return to sport or knee function.

**Level of evidence:**

III.

**Trial registration:**

https://clinicaltrials.gov/, ClinicalTrials.gov Identifier: NCT02511158

## Introduction

According to the meta-analysis by Bansal et al. [1], the incidence of septic arthritis of the knee following primary ACL reconstruction with an autograft is 0.19% (95% CI 0.03–0.43%). The clinical results in patients with septic arthritis are poorer than in those without infection, whatever the length of follow-up [3, 4, 18, 20].

Several recent studies have shown that vancomycin-soaked grafts are effective in preventing infection [5, 13–15, 19, 21]. All published studies evaluating this subject have confirmed that none of the patients developed septic arthritis when a vancomycin-soaked graft was used. The meta-analysis by Naendrup et al. [12] based on four comparative studies, including 5076 patients and 2976 who received a vancomycin-soaked graft, found an odds ratio of 0.04 [0.01–0.16] in favour of the vancomycin group.

But is this technique safe [6, 9]? One in vitro study in porcine tendons did not find any change in the biomechanical properties of tendons when vancomycin-soaked compresses were applied at concentrations of between 1 and 10 mg/ml for 10 and 20 min [17]. Certain earlier clinical studies have shown that soaking does not compromise function. The evaluation criteria in these studies were the rate of retears [13, 21], functional scores (subjective International Knee Documentation Committee [IKDC] [13], Tegner [5, 13] and Lysholm [5] scores) or the return to the same preinjury activity level [5]. However, these studies included the general population and they did not evaluate the return to sport.

The main goal of this study was to compare return to sport and knee function 1 year after ACL reconstruction in athletes with and without grafts presoaked in a vancomycin solution. The hypothesis of the study was that the return-to-sport rate in the group with vancomycin-soaked grafts was not inferior to that in the group without vancomycin-soaked grafts.

## Materials and methods

The study was approved by an ethics committee (Comité de Protection des Personnes Ile-de-France VI, Hôpital La Pitié Salpêtrière), and patient consent was obtained. This single-centre prospective cohort study began in 2012 and included all patients who underwent reconstruction for an ACL tear by four senior surgeons in a centre specialized in sports surgery. A case–control study was performed based on a retrospective analysis of prospective data including a continuous series of patients ≥ 16 years old operated between 2012 and 2018 for a primary or recurrent ACL tear, associated or not with reconstruction of lateral ligaments. Exclusion criteria were posterior cruciate ligament tears, isolated lateral ligament tears, tibial spine avulsion fractures and missing data on the return to sport after 1 year of follow-up. Because of a change in protocols, all patients operated after 1 November 2016 received a graft soaked in a vancomycin solution. Two groups of patients were evaluated: Group 1 « without vancomycin group» before November 2016 and Group 2 « with vancomycin group» after that date.

### Study protocols

#### Antibiotic protocol

The same antibiotic protocol was followed in all patients. Thirty min before applying a pneumatic tourniquet, 2 g of cephalothin was administered intravenously. In case of allergies, the patient received 1 g of vancomycin. This injection was repeated 2 h later for longer surgeries.

#### Protocol for autograft vancomycin soaking

The vancomycin solution was prepared outside the surgical field according to the protocol described by Grayson et al. [7]. The solution was then poured into a dish placed on the instrument table in the surgical field using best practice aseptic techniques. Once the autograft had been harvested, it was thoroughly soaked in the vancomycin solution for 10 min; then, the graft was fixed in the tibial and femoral tunnels.

#### ACL reconstruction surgical protocol

ACL reconstruction was performed using spinal or general anaesthesia depending on the patient’s wishes and the surgeon’s usual practices. A tourniquet was always used. An arthroscopic approach was taken with a hamstring tendon (semitendinosus, gracilis), patellar tendon or the tensor fasciae latae tendon graft. Extraarticular reconstruction of the fascia latae was associated with the procedure if the surgeon considered it necessary for knee stability [11]. The aim of this technique is to perform anatomical reconstruction of the anterolateral ligament using the iliotibial band to improve control of anterior laxity and medial rotation of the tibia.

#### Management of septic arthritis

Emergency arthroscopic debridement and lavage were performed. The grafts were preserved, and none of the material was changed. Patients received empirical intravenous post-operative antibiotics which were then adapted when the etiological agent was determined by antibiogram. Antibiotic treatment lasted a total of 6 weeks including intravenous antibiotics for 10 days [3].

### Data collection

Patients were all contacted by email for each follow-up assessment and sent a link to an electronic version of the follow-up questionnaire. For the return to sport, patients were asked “Have you returned to running?”, “Have you returned to playing the sport you practiced before your injury?” and “If yes, do you think you are playing at a lower, the same or a higher level as before your injury?”. The online questionnaire was constructed and administered by the software WebSurvey^®^. In the absence of a response, a second email was sent to patients and if necessary, he/she was contacted by telephone.

### Evaluation criteria

The main evaluation criterion was the return to sport 1 year after surgery based on the return to running and the return to the preinjury sport.

Secondary evaluation criteria were complications, defined as any adverse event related to surgery in the first year of follow-up, functional knee scores (IKDC [8], knee injury and osteoarthritis outcome score [KOOS] [16] and ACL-return to sports after injury [ACL-RSI] scores [2]) and patient satisfaction (very satisfied/satisfied/fairly satisfied/not satisfied) at 1-year follow-up.

### Statistical analysis

The number of patients needed for a non-inferiority study was calculated with the statistical software XLSTAT. According to a recent meta-analysis [10], the return-to-sport rate after ACL reconstruction surgery is 83% (95% CI 77–88%). The calculated hypotheses were a non-inferiority margin of 10% in the percentage of return to sport after 1 year, for the range of the confidence interval, an expected rate of return to sport of 83%, a power of 0.80 and an alpha risk of 0.025. A minimum of two hundred and twenty-one patients were needed per group. Normal distribution was tested by the Shapiro–Wilk test. The Student t test was used for quantitative variables and the Chi-square test for qualitative variables. The nonparametric Mann and Whitney test was performed in small groups for quantitative variables and the Fischer exact test for qualitative variables. Factors associated with the return to the preinjury sport at the 1-year follow-up were identified on univariate analysis and then multivariate analysis by logistic regression. Covariates were selected based on the results of univariate analysis (selecting only factors with a *p* value < 0.2) and any known potential causal relationships between factors to avoid overadjustment. A *p* value < 0.05 was considered to be statistically significant.

## Results

### Study flow chart at inclusion

**Fig. 1:**
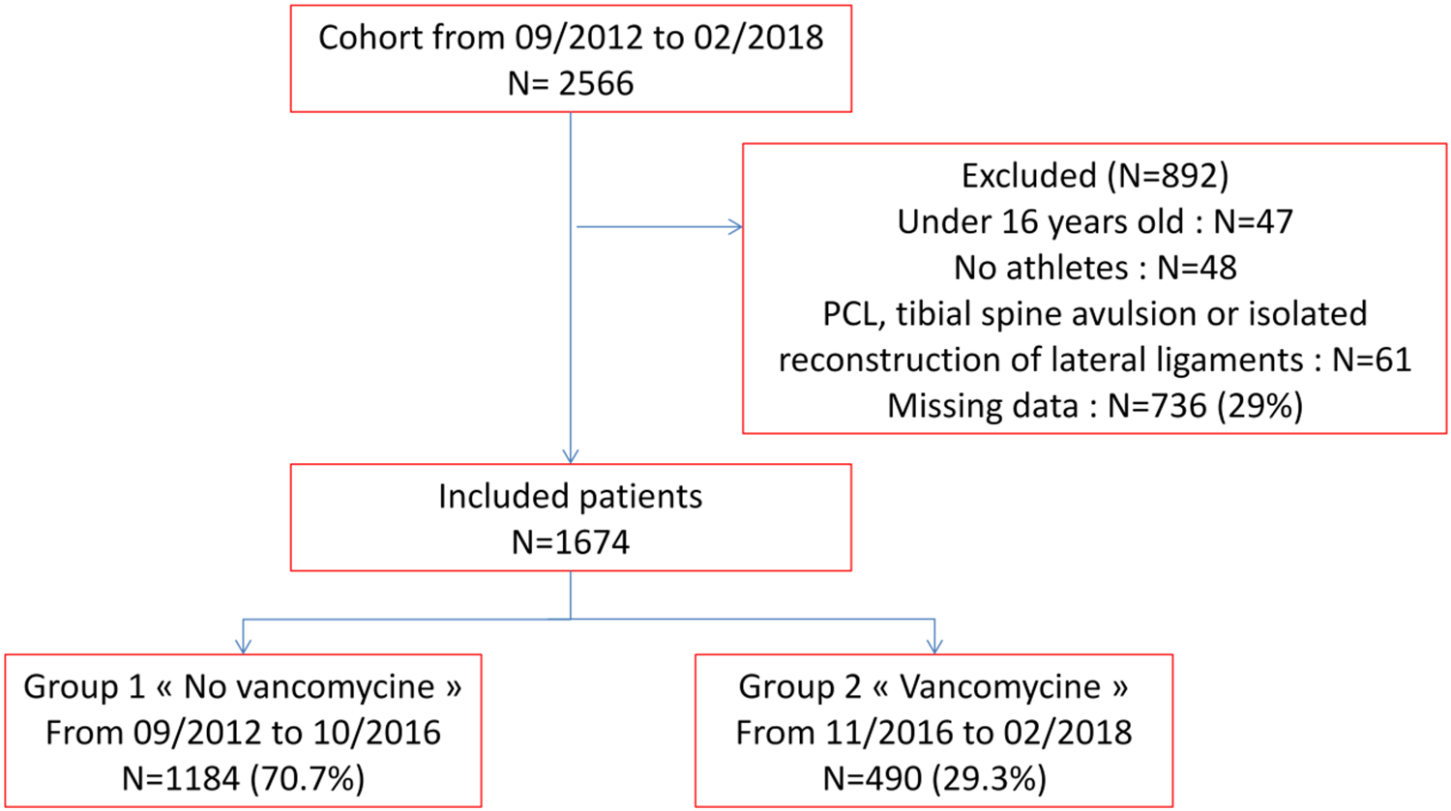
Study flow chart at inclusion

A total of 1674 patients fulfilled the selection criteria during the study period, 1184 (70.7%) in Group 1 and 490 (29.3%) in Group 2 (Fig. 1).

### Description of the study population

The two groups were comparable at inclusion (Table 1).

**Table 1:** Demographic and clinical data before ACL reconstruction

	Group 1 (*N* = 1184)	Group 2 (*N* = 490)	*p* value
Age (years)	29.9 ± 9.6	30.3 ± 9.9	n.s.
Sex	Male 794 (67.1%)	Male 318 (64.9%)	n.s.
	Female 390 (32.9%)	Female 172 (35.1%)	
BMI	24 ± 3.3	23.8 ± 3.3	n.s.
Level of sport	Professional 45 (3.8%)	23	n.s.
	Competitive 481 (40.6%)	193	
	Regular leisure 505 (42.7%)	217	
	Occasional leisure 153 (12.9%)	57	
Type of preinjury sport	Pivot contact 735 (62.1%)	296 (60.4%)	n.s.
	Pivot without contact 280 (23.6%)	126 (25.7%)	
	Without pivot 167 (14.1%)	68 (13.9%)	
	NR* 2 (0.2%)	0	
History of knee surgery**	137 (11.6%)	60 (12.2%)	n.s.
IKDC subjective	59.3 ± 16.5	58.9 ± 17.3	n.s.
KOOS symptoms/stiffness	72.5 ± 17.5	71.6 ± 18.8	n.s.
KOOS pain	75.8 ± 16.7	74.1 ± 17.8	n.s.
KOOS daily life	84.7 ± 16.4	82.8 ± 18.3	0.04
KOOS sport	47.7 ± 28.1	44.4 ± 28.7	n.s.
KOOS quality of life	30.9 ± 21.3	29.7 ± 21.7	n.s.
ACL-RSI	40.2 ± 26.3	40.8 ± 26.1	n.s.
Lysholm	71 ± 17.7	69.2 ± 18.5	n.s.
GNRB laxity at 200 N (mm)	3.5 ± 2.6	3.3 ± 1.9	n.s.

*Not reported, **Revision surgery, prior meniscal or cartilage surgery

Surgical data (Table 2) show that reconstruction using the tensor fascia latae tendon (*p* < 10^−4^) and extraarticular reconstruction (*p* < 10^−4^) were significantly more frequent in Group 2. Patients in Group 1 more frequently presented with a cartilage defect that was identified perioperatively (*p* = 0.02), as well as with a medial (*p* < 10^−4^) or lateral (*p* < 10^−4^) meniscal injury, than those in Group 2.

**Table 2:** Surgical data for ACL reconstruction

	Group 1 (*N* = 1184)	Group 2 (*N* = 490)	*p* value
Type of graft	HS^(1)^ 977 (82.5%)	352 (71.8%)	< 10^−4^
	BPTB^(2)^ 115 (9.7%)	38 (7.8%)	
	CFL^(3)^ 92 (7.8%)	100 (20.4%)	
Extraarticular tenodesis (FLT^(4)^)	446 (37.7%)	234 (47.7%)	< 10^−4^
Chondropathy	239 (20.2%)	76 (15.5%)	0.02
Medial meniscal lesion	401 (33.9%)	104 (21.2%)	< 10^−4^
Lateral meniscal lesion	350 (29.6%)	98 (20%)	< 10^−4^

^(1)^Hamstring tendon, ^(2)^Bone–patellar tendon–bone, ^(3)^Combined fasciae latae for intra- and extraarticular anterior cruciate ligament reconstruction, ^(4)^Tensor fasciae latae tendon

### Complications during post-operative year one

In the first week, 74 (4.4%) patients in the series presented with one or more complications. Complications were significantly more frequent in Group 1 (5.2% vs. 2.5%, *p* = 0.01) (Table 3). Septic arthritis of the operated knee developed in seven patients, all in Group 1, after between 7 and 21 days (Table 3). The causative agent was always a staphylococcus: 4 *S.epidermidis MS* (*Methicillin*-*Sensitive*) associated in one case with *Propionibacterium acnes*, 1 *S.capitis MR (Methicillin*-*Resistant),* 1 *S.capitis MS* and 1 *S.aureus MS.*

**Table 3:** Complications during post-operative year one

	Group 1 (*N* = 1184)	Group 2 (*N* = 490)	*p* value
Early complications in the first week after surgery (78 in 74 patients)			
Diffuse haematomas in the popliteal fossa	49 (4.1%)	7 (1.4%)	0.004
Hemarthroses^(1)^	9 (0.8%)	2 (0.4%)	n.s.
Stiff knee^(2)^	4 (0.3%)	2 (0.4%)	n.s.
Phlebitis	3 (0.2%)	0	n.s.
Others	2^(3)^ (0.2%)	0	n.s.
Secondary complications in post-operative year one (60 in 57 patients)			
Septic arthritis	7 (0.6%)	0	0.08
Recurrent tear	10 (0.8%)	0	0.04
Cyclops lesion	21 (1.8%)	3 (0.6%)	0.07
Meniscal surgery^(4)^	9 (0.8%)	0	0.06
Cartilage surgery^(5)^	2 (0.2%)	2 (0.4%)	n.s.
Knee surgery^(6)^	1 (0.1%)	3 (0.6%)	0.07
Other^(7)^	1 (0.1%)	1 (0.2%)	n.s.

^(1)^8 required simple puncture or surgical draining, ^(2)^3 required mobilization under general anaesthesia, ^(3)^1 algodystrophy and 1 blistering of the scar, ^(4)^Meniscectomies or suture, ^(5)^Microfractures or chondrocyte graft, ^(6)^All requiring mobilization under general or locoregional anaesthesia, ^(7)^1 transosseous reinsertion of the patellar ligament and 1 repair of the scar

In the first year, 50 (3%) patients presented with at least one complication in the operated knee besides infection, 5.9 ± 3.3 months after surgery with no significant difference between the two groups (n.s.). It should be noted that all retears occurred in Group 1 (Table 3).

Seven (0.4%) patients presented with a contralateral ACL tear in the first year, six in Group 1 and one in Group 2, (0.5% vs. 0.2%, n.s.).

### Main evaluation criteria

At the 1-year follow-up, there was no significant difference between the two groups for the return to running (Table 4). However, significantly more patients in Group 2 returned to their preinjury sport (Table 4).

**Table 4:** Criteria for return to sport at 1 year of follow-up

	Group 1 (*N* = 1184)	Group 2 (*N* = 490)	*p* value
Return to running	901 (76.1%)	372 (75.9%)	n.s.
Delay (months)	6.9 ± 3.3	6.4 ± 2.6	0.01
Return to usual preinjury sport	618 (52.2%)	282 (57.5%)	0.04
Delay (in months)	9.2 ± 3.1	8.9 ± 2.6	n.s.
Level of play in case of return to same preinjury sport	Higher 31 (5%)	18 (6.4%)	n.s.
	Same 246 (39.8%)	117 (41.5%)	
	Lower 330 (53.4%)	143 (50.7%)	
	Not reported 11 (1.8%)	4 (1.4%)	

### Functional knee scores at 1 year of follow-up and patient satisfaction

At the 1-year follow-up, Group 2 presented with significantly better KOOS symptom and stiffness scores. There was no significant difference in the other functional knee scores or in patient satisfaction (Table 5).

**Table 5:** Functional knee scores and patient satisfaction at 1 year of follow-up

	Group 1 (*N* = 1184)	Group 2 (*N* = 490)	*p* value
Subjective IKDC (/100)	82.1 ± 13.5	82.7 ± 13.7	n.s.
KOOS symptoms/stiffness (/100)	78.9 ± 15.9	84 ± 13.6	< 10^−4^
KOOS pain (/100)	89.7 ± 10.8	89.9 ± 10.6	n.s.
KOOS daily life (/100)	95.7 ± 8.5	96 ± 8.5	n.s.
KOOS sport (/100)	78.5 ± 20.3	79.4 ± 20.5	n.s.
KOOS quality of life (/100)	68.1 ± 22.5	69.1 ± 20.3	n.s.
Lysholm (/100)	88.5 ± 11.5	88.8 ± 12.6	n.s.
ACL-RSI (/100)	64.3 ± 24.1	64.5 ± 22.6	n.s.
Very satisfied and satisfied	1074 (90.7%)	452 (92.2%)	n.s.

### Factors favouring the return to the preinjury sport

Univariate analysis showed that compared to patients who did not return to their preinjury sport at the 1-year follow-up, patients who did return to their preinjury sport were significantly younger and lighter, were professional or competitive athletes without a prior history of knee surgery, had better preoperative functional and psychological scores (subjective IKDC, KOOS daily life, sports and quality of life, Lysholm and ACL-RSI), belonged to the “with vancomycin” group, underwent associated extraarticular reconstruction, presented with fewer medial meniscal lesions, and did not present with complications during the first year after surgery, in particular septic arthritis (Table 6).

**Table 6:** Factors favouring the return to the preinjury sport at 1 year of follow-up

	Return to preinjury sport (*N* = 900)	No return to preinjury sport (*N* = 774)	*p* value
Age (years)	29 ± 9.7	31.2 ± 9.6	< 10^−4^
Male gender	612 (68%)	500 (64.6%)	n.s.
BMI	23.7 ± 3.1	24.2 ± 3.5	0.001
Competitive or professional athlete	493 (54.8%)	249 (32.2%)	0.0001
History of knee surgery	88 (9.8%)	109 (14.1%)	0.006
Preoperative subjective IKDC (/100)	60.6 ± 16.4	57.6 ± 16.9	0.0002
Preoperative KOOS symptoms/stiffness (/100)	73 ± 17	71.5 ± 18.8	n.s.
Preoperative KOOS pain (/100)	75.9 ± 16.6	74.6 ± 17.6	n.s.
Preoperative KOOS daily life (/100)	85.1 ± 16.4	82.9 ± 17.7	0.008
Preoperative KOOS sport (/100)	47.4 ± 28.3	42.9 ± 28.1	0.001
Preoperative KOOS quality of life (/100)	32.7 ± 22	28.1 ± 20.5	< 10^−4^
Preoperative Lysholm (/100)	72.1 ± 17.8	68.6 ± 18	0.0001
Preoperative ACL-RSI (/100)	43.8 ± 26.8	36.6 ± 25.1	< 10^−4^
Associated extraarticular reconstruction	390 (43.3%)	290 (37.5%)	0.01
Vancomycin soaking	282 (31.3%)	208 (26.9%)	0.04
Chondropathy	154 (17.1%)	161 (20.8%)	n.s.
Medial meniscal lesion	237 (26.3%)	268 (34.6%)	0.0001
Lateral meniscal lesion	248 (27.6%)	200 (25.8%)	n.s.
Post-operative septic arthritis	1 (0.1%)	6 (0.8%)	0.03
Development of a complication besides infection during post-operative year one	48 (5.3%)	74 (9.6%)	0.001

The final multivariate analysis included the study group (1 or 2), age under 25, male gender, competitive or professional sport, a history of knee surgery, preoperative scores (subjective IKDC ≥ 65, KOOS daily life ≥ 85, KOOS sport ≥ 70, KOOS quality of life ≥ 40, ACL-RSI ≥ 55), the association of extraarticular reconstruction, the presence of a medial meniscal tear, the development of a severe complication during the first year (including an infection and a homolateral retear). The preoperative Lysholm score was not included in this model because it was strongly correlated with the subjective IKDC score. Six variables were significantly correlated with the return to the preinjury sport at the 1-year follow-up on multivariate analysis. The factors favouring the return to the preinjury sport were practicing a professional or competitive sport (OR = 2.5 [2–3.1], *p* < 10^−4^), a preoperative subjective IKDC ≥ 65 (OR = 1.3 [1.1–1.6], *p* = 0.01) and a preoperative ACL-RSI score ≥ 55 (OR = 1.5 [1.2–1.9], *p* = 0.001). The risk factors of not returning to the preinjury sport included a history of knee surgery (OR = 0.7 [0.5–0.9], *p* = 0.009), the presence of a medial meniscal lesion (OR = 0.7 [0.6–0.9], *p* = 0.01) and the development of a severe complication during post-operative year one (OR = 0.5 [0.3–0.7], *p* < 10^−4^).

## Discussion

The main finding of this case–control study was that the return to sport following ACL reconstruction was not influenced by vancomycin presoaking of the graft. The hypothesis of non-inferiority was confirmed. After adjustment for all statistically significant variables, the patients who returned to their preinjury sport in this study were professional or competitive athletes with good preoperative functional and psychological scores, no history of surgery, no medial meniscal injuries and no severe complications in the first year after surgery. Vancomycin soaking of the graft was not associated with the return to sport on univariate or multivariate analysis.

Like all previously published studies on the subject, there was no septic arthritis reported in the knees in the “with vancomycin” group. Moreover, all retears occurred in the “without vancomycin” group, or 0.8% of the patients in this group at 1 year of follow-up. Vertullo et al. [21] identified a retear rate of 4.6% in the “without vancomycin” group at the 5.8-year follow-up and 2.75% in the “with vancomycin group” at the 2.6-year follow-up. For Offerhaus et al. [13], this rate was 10% in the group “without vancomycin” and 3% in the group “with vancomycin”. Thus, vancomycin presoaking does not increase the risk of recurrent tears. There was no significant difference found between the two groups for the development of serious complications in the first year after surgery except for infection and homolateral retears.

The knee scores were comparable in the two groups at 1-year follow-up, which is similar to results in prior studies. In our study, the KOOS stiffness score was significantly better in the vancomycin group. Although Offerhaus et al. [13] found more stiffness in the vancomycin group (11% vs. 7%), this difference was not statistically significant. Brandl et al. [5] did not find any significant difference in the level of post-operative activity between the two groups compared to the level of preinjury activity.

The protocols of vancomycin autograft presoaking are very similar in all the published studies. All of these studies were based on the protocol described by Grayson et al. [7]. In our study, the transplant was soaked in a dish containing a vancomycin solution for 10 min and then placed and fixed in the bone tunnel without rinsing. Other teams wrapped the graft in compresses soaked in this solution for 10–20 min, and some rinsed it with saline solution before graft placement.

This study confirms that soaking an autograft in vancomycin is effective against the risk of infection and also provides other specific elements of safety to athletes. The meta-analysis performed on this subject has confirmed the value of this technique as well [12]. Vancomycin presoaking of the graft during ACL reconstruction should therefore be recommended during ACL reconstruction according to grade C of the Oxford Centre for Evidence-Based Medicine, based on existing studies, including the present study, with a level of evidence of 3 or 4. Because the incidence of this complication is very low, it would be difficult to organize randomized studies on this topic.

The present study has certain limitations. First, it was not a randomized study. However, the number of patients needed was calculated for a non-inferiority study on the return to sport, guaranteeing its statistical power. Moreover, inclusion of all the patients in the cohort who fulfilled the selection criteria provided a more exact incidence of septic arthritis and rate of recurrent tears. Although a 1-year follow-up may seem short, the inclusion period was long, allowing us to evaluate all patients for the same length of follow-up.

In day-by-day practice, vancomycin presoaking of the graft during ACL reconstruction should be recommended during ACL reconstruction because it reduces the risk of post-operative knee infection without compromising function.

## Conclusion

Vancomycin presoaking of autografts during ACL reconstruction reduces the risk of post-operative infection without affecting return to sport or knee function.
