# Correction to: Vancomycin-soaked autografts during ACL reconstruction reduce the risk of post-operative infection without affecting return to sport or knee function

**DOI:** 10.1007/s00167-020-05923-8

**Published:** 2020-03-06

**Authors:** Yoann Bohu, Shahnaz Klouche, Hasan Basri Sezer, Serge Herman, Olivier Grimaud, Antoine Gerometta, Alain Meyer, Nicolas Lefevre

**Affiliations:** 1 Clinique du Sport Paris, 36 Boulevard Saint Marcel, 75005 Paris, France; 2 Racing 92, 11 Avenue du Plessis, 92350 Plessis-Robinson, France; 3 Institut de l’Appareil Locomoteur Nollet, 23, Rue Brochant, 75013 Paris, France; 4 ELSAN, 58bis Rue de la Boétie, 75008 Paris, France

## Correction to: Knee Surgery, Sports Traumatology, Arthroscopy 10.1007/s00167-020-05879-9

The article Vancomycin‑soaked autografts during ACL reconstruction reduce the risk of post‑operative infection without affecting return to sport or knee function, written by Yoann Bohu, Shahnaz Klouche, Hasan Basri Sezer, Serge Herman, Olivier Grimaud, Antoine Gerometta, Alain Meyer, Nicolas Lefevre was originally published electronically on the publisher’s internet portal on 05 February 2020 without open access. With the author(s)’ decision to opt for Open Choice the copyright of the article changed on 06 March 2020 to © The Author(s) 2020 and the article is forthwith distributed under a Creative Commons Attribution 4.0 International License (https://creativecommons.org/licenses/by/4.0/), which permits use, sharing, adaptation, distribution and reproduction in any medium or format, as long as you give appropriate credit to the original author(s) and the source, provide a link to the Creative Commons licence, and indicate if changes were made.

The original article has been corrected.

